# *N*′-[(3-Methyl-2-thien­yl)carbon­yl]isonicotinohydrazide

**DOI:** 10.1107/S1600536809033030

**Published:** 2009-08-26

**Authors:** H. S. Naveenkumar, Amirin Sadikun, Pazilah Ibrahim, Jia Hao Goh, Hoong-Kun Fun

**Affiliations:** a School of Pharmaceutical Sciences, Universiti Sains Malaysia, 11800 USM, Penang, Malaysia; b X-ray Crystallography Unit, School of Physics, Universiti Sains Malaysia, 11800 USM, Penang, Malaysia

## Abstract

In the title compound, C_12_H_11_N_3_O_2_S, the pyridine ring is inclined to the thio­phene ring, forming a dihedral angle of 34.96 (7)°. The mean plane through the hydrazide unit forms dihedral angles of 21.57 (8) and 53.08 (8)°, respectively, with the pyridine and thio­phene rings. The two O atoms are twisted away from each other, as indicated by the C—N—N—C torsion angle of −81.27 (15)°. In the crystal structure, mol­ecules are linked into an extended three-dimensional network by inter­molecular N—H⋯N, N—H⋯O and C—H⋯O hydrogen bonds. The crystal structure also features a short S⋯O [3.2686 (10) Å] inter­action and a weak inter­molecular C—H⋯π inter­action.

## Related literature

For general background to and application of isoniazid derivatives, see: Janin (2007 ▶); Maccari *et al.* (2005 ▶); Slayden *et al.* (2000 ▶). For the preparation of the title compound, see: Besra *et al.* (1993 ▶). For bond-length data, see: Allen *et al.* (1987 ▶). For a closely related structure, see: Naveenkumar *et al.* (2009 ▶). For the stability of the temperature controller used for the data collection, see: Cosier & Glazer (1986 ▶).
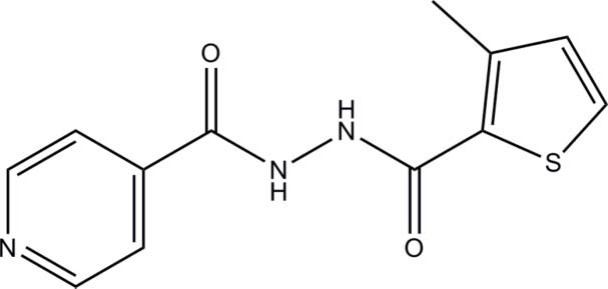


## Experimental

### 

#### Crystal data

C_12_H_11_N_3_O_2_S
*M* *_r_* = 261.30
Orthorhombic, 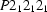

*a* = 8.9206 (1) Å
*b* = 10.7552 (2) Å
*c* = 12.4934 (2) Å
*V* = 1198.65 (3) Å^3^
*Z* = 4
Mo *K*α radiation
μ = 0.27 mm^−1^
*T* = 100 K
0.58 × 0.20 × 0.15 mm

#### Data collection

Bruker SMART APEXII CCD area-detector diffractometer
Absorption correction: multi-scan (**SADABS**; Bruker, 2005 ▶) *T*_min_ = 0.861, *T*_max_ = 0.961
17333 measured reflections
4355 independent reflections
4078 reflections with *I* > 2σ(*I*)
*R*_int_ = 0.025

#### Refinement

*R*[*F* ^2^ > 2σ(*F* ^2^)] = 0.036
*wR*(*F* ^2^) = 0.097
*S* = 1.05
4355 reflections
165 parameters
H-atom parameters constrained
Δρ_max_ = 0.79 e Å^−3^
Δρ_min_ = −0.43 e Å^−3^
Absolute structure: Flack (1983 ▶), 1856 Friedel pairs
Flack parameter: −0.04 (6)

### 

Data collection: *APEX2* (Bruker, 2005 ▶); cell refinement: *SAINT* (Bruker, 2005 ▶); data reduction: *SAINT*; program(s) used to solve structure: *SHELXTL* (Sheldrick, 2008 ▶); program(s) used to refine structure: *SHELXTL*; molecular graphics: *SHELXTL*; software used to prepare material for publication: *SHELXTL* and *PLATON* (Spek, 2009 ▶).

## Supplementary Material

Crystal structure: contains datablocks global, I. DOI: 10.1107/S1600536809033030/is2451sup1.cif

Structure factors: contains datablocks I. DOI: 10.1107/S1600536809033030/is2451Isup2.hkl

Additional supplementary materials:  crystallographic information; 3D view; checkCIF report

## Figures and Tables

**Table 1:** Hydrogen-bond geometry (Å, °)

*D*—H⋯*A*	*D*—H	H⋯*A*	*D*⋯*A*	*D*—H⋯*A*
N2—H1*N*2⋯N1^i^	0.86	2.14	2.9068 (17)	149
N3—H1*N*3⋯O2^ii^	0.86	1.99	2.8034 (15)	158
C4—H4*A*⋯O1^iii^	0.93	2.58	3.2047 (17)	125
C10—H10*A*⋯O2^iv^	0.93	2.51	3.3928 (19)	159
C11—H11*A*⋯*Cg*1^v^	0.93	2.80	3.3760 (17)	121

Symmetry codes: (i) 
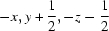
; (ii) 
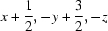
; (iii) 
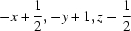
; (iv) 
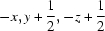
; (v) 
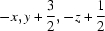
. *Cg*1 is centroid of the C1/C2/N1/C3–C5 ring.

**Fig. 1.**
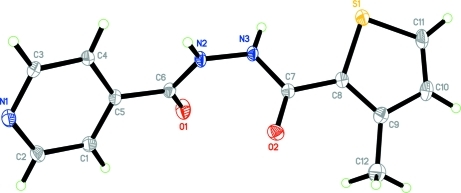
The molecular structure of the (I), showing 50% probability displacement ellipsoids and the atom-numbering scheme. Hydrogen atoms are shown as spheres of arbitrary radius.

**Fig. 2.**
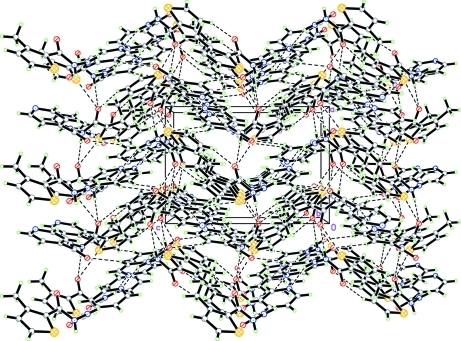
Three dimensional extended network, viewed along the b axis. Intermolecular interactions are shown as dashed lines.

## supplementary crystallographic information

### Comment

In the search of new compounds, isoniazid derivatives have been found to possess potential tuberculostatic activity (Janin, 2007; Maccari *et al.*, 2005; Slayden *et al.*, 2000). As a part of a current work on synthesis of such derivatives, in this paper we present the crystal structure of the title compound, (I) which was synthesized in our lab.

In (I), the pyridine ring (C1/C2/N1/C3-C5) is inclined to the thiophene ring (C8-C11/S1), forming a dihedral angle of 34.96 (7)° (Fig. 1). The mean plane through the hydrazide unit (C7/N2/N3/O2) forms dihedral angles of 21.57 (8) and 53.08 (8)°, respectively, with the pyridine and thiophene rings. The O1 and O2 atoms are twisted away from each other as indicated by torsion angle C6–N2–N3–C7 [-81.27 (15)°]. The bond lengths (Allen *et al.*, 1987) and angles are comparable to a closely related structure (Naveenkumar *et al.*, 2009).

In the crystal structure (Fig. 2), the molecules are linked into an extended three-dimensional network by intermolecular C4—H4A···O1, C10—H10A···O2, N2—H1N2···N1 and N3—H1N3···O2 hydrogen bonds (Table 1). The crystal structure is further stabilized by short S1···O2 interactions of 3.2686 (10) Å [symmetry code: 1/2+x, 3/2-y, -z] and by weak intermolecular C11—H11A···*Cg*1 interactions (Table 1; *Cg*1 is the centroid of the pyridine ring).

### Experimental

Compound (I) was prepared following the procedure by literature (Besra *et al.*, 1993). Dry dichloromethane (30 ml) and 4-dimethylaminopyridine (4-DMAP) (1.2 eq) was added to 3-methylthiophene-2-carbonyl chloride followed by isoniazid (1.1 eq). The reaction mixture was kept in an ice bath for 1 h and then left stirring under nitrogen atmosphere overnight at room temperature. Dichloromethane (20 ml) was added to the reaction mixture, which was then washed with water, and the organic layer dried over anhydrous sodium sulphate. The solvent was removed under reduced pressure to afford the crude product which was purified by column chromatography and recrystallized from ethanol to afford colorless single crystals.

### Refinement

All the H atoms were placed in calculated positions, with N—H = 0.86 Å, *U*_iso_(H) = 1.2 *U*_eq_(N), C—H = 0.93 Å, *U*_iso_(H) = 1.2 *U*_eq_(C) for aromatic, and C—H = 0.96 Å, *U*_iso_(H) = 1.5 *U*_eq_(C) for methyl group. These H atoms were refined as riding on their parent atoms. A rotating group model was used for the methyl group.

### Crystal data

**Table:**

C_12_H_11_N_3_O_2_S	*F*(000) = 544
*M**_r_* = 261.30	*D*_x_ = 1.448 Mg m^−^^3^
Orthorhombic, *P*2_1_2_1_2_1_	Mo *K*α radiation, λ = 0.71073 Å
Hall symbol: P 2ac 2ab	Cell parameters from 9634 reflections
*a* = 8.9206 (1) Å	θ = 2.5–32.7°
*b* = 10.7552 (2) Å	µ = 0.27 mm^−^^1^
*c* = 12.4934 (2) Å	*T* = 100 K
*V* = 1198.65 (3) Å^3^	Block, colourless
*Z* = 4	0.58 × 0.20 × 0.15 mm

### Data collection

**Table:**

Bruker SMART APEXII CCD area-detector diffractometer	4355 independent reflections
Radiation source: fine-focus sealed tube	4078 reflections with *I* > 2σ(*I*)
graphite	*R*_int_ = 0.025
φ and ω scans	θ_max_ = 32.7°, θ_min_ = 2.5°
Absorption correction: multi-scan (*SADABS*; Bruker, 2005)	*h* = −13→13
*T*_min_ = 0.861, *T*_max_ = 0.961	*k* = −14→16
17333 measured reflections	*l* = −17→18

### Refinement

**Table:**

Refinement on *F*^2^	Secondary atom site location: difference Fourier map
Least-squares matrix: full	Hydrogen site location: inferred from neighbouring sites
*R*[*F*^2^ > 2σ(*F*^2^)] = 0.036	H-atom parameters constrained
*wR*(*F*^2^) = 0.097	*w* = 1/[σ^2^(*F*_o_^2^) + (0.0542*P*)^2^ + 0.3319*P*] where *P* = (*F*_o_^2^ + 2*F*_c_^2^)/3
*S* = 1.05	(Δ/σ)_max_ < 0.001
4355 reflections	Δρ_max_ = 0.79 e Å^−^^3^
165 parameters	Δρ_min_ = −0.43 e Å^−^^3^
0 restraints	Absolute structure: Flack (1983), 1856 Friedel pairs
Primary atom site location: structure-invariant direct methods	Flack parameter: −0.04 (6)

### Special details

**Table:**

Experimental. The crystal was placed in the cold stream of an Oxford Cyrosystems Cobra open-flow nitrogen cryostat (Cosier & Glazer, 1986) operating at 100.0 (1)K.
Geometry. All esds (except the esd in the dihedral angle between two l.s. planes) are estimated using the full covariance matrix. The cell esds are taken into account individually in the estimation of esds in distances, angles and torsion angles; correlations between esds in cell parameters are only used when they are defined by crystal symmetry. An approximate (isotropic) treatment of cell esds is used for estimating esds involving l.s. planes.
Refinement. Refinement of F^2^ against ALL reflections. The weighted R-factor wR and goodness of fit S are based on F^2^, conventional R-factors R are based on F, with F set to zero for negative F^2^. The threshold expression of F^2^ > 2sigma(F^2^) is used only for calculating R-factors(gt) etc. and is not relevant to the choice of reflections for refinement. R-factors based on F^2^ are statistically about twice as large as those based on F, and R- factors based on ALL data will be even larger.

### Fractional atomic coordinates and isotropic or equivalent isotropic displacement parameters (Å^2^)

**Table:**

	*x*	*y*	*z*	*U*_iso_*/*U*_eq_	
S1	0.26735 (4)	0.98879 (3)	0.04525 (3)	0.01858 (8)	
O1	0.29108 (13)	0.48575 (10)	0.02488 (8)	0.0221 (2)	
O2	0.01066 (11)	0.69442 (9)	0.07046 (8)	0.01664 (19)	
N1	0.03034 (14)	0.21406 (11)	−0.25010 (10)	0.0159 (2)	
N2	0.18343 (13)	0.62066 (10)	−0.09379 (9)	0.0137 (2)	
H1N2	0.1404	0.6288	−0.1550	0.016*	
N3	0.21647 (13)	0.72506 (10)	−0.03274 (9)	0.01367 (19)	
H1N3	0.2954	0.7684	−0.0458	0.016*	
C1	0.11893 (17)	0.29049 (12)	−0.08076 (11)	0.0165 (2)	
H1A	0.1343	0.2764	−0.0081	0.020*	
C2	0.05396 (17)	0.19936 (13)	−0.14457 (11)	0.0174 (2)	
H2A	0.0255	0.1248	−0.1128	0.021*	
C3	0.07732 (15)	0.32069 (12)	−0.29452 (11)	0.0153 (2)	
H3A	0.0653	0.3306	−0.3680	0.018*	
C4	0.14288 (15)	0.41719 (12)	−0.23723 (11)	0.0143 (2)	
H4A	0.1741	0.4894	−0.2716	0.017*	
C5	0.16079 (15)	0.40323 (12)	−0.12698 (10)	0.0136 (2)	
C6	0.21924 (14)	0.50531 (11)	−0.05670 (10)	0.0138 (2)	
C7	0.12212 (14)	0.75769 (11)	0.04814 (11)	0.0125 (2)	
C8	0.15680 (15)	0.87488 (12)	0.10399 (11)	0.0142 (2)	
C9	0.10620 (16)	0.91137 (14)	0.20356 (11)	0.0177 (2)	
C10	0.15852 (18)	1.03210 (14)	0.23047 (13)	0.0209 (3)	
H10A	0.1356	1.0716	0.2947	0.025*	
C11	0.24567 (18)	1.08445 (13)	0.15288 (12)	0.0214 (3)	
H11A	0.2883	1.1631	0.1581	0.026*	
C12	0.0117 (2)	0.83596 (16)	0.27738 (13)	0.0259 (3)	
H12A	0.0530	0.7538	0.2835	0.039*	
H12B	−0.0884	0.8309	0.2496	0.039*	
H12C	0.0097	0.8745	0.3466	0.039*	

### Atomic displacement parameters (Å^2^)

**Table:**

	*U*^11^	*U*^22^	*U*^33^	*U*^12^	*U*^13^	*U*^23^
S1	0.02092 (15)	0.01399 (13)	0.02083 (16)	−0.00198 (11)	0.00366 (12)	−0.00283 (12)
O1	0.0302 (5)	0.0186 (4)	0.0176 (5)	0.0033 (4)	−0.0104 (4)	−0.0021 (4)
O2	0.0134 (4)	0.0166 (4)	0.0200 (5)	−0.0026 (3)	0.0000 (3)	0.0000 (4)
N1	0.0187 (5)	0.0131 (5)	0.0160 (5)	−0.0004 (4)	−0.0017 (4)	−0.0018 (4)
N2	0.0177 (5)	0.0113 (4)	0.0120 (5)	−0.0003 (4)	−0.0028 (4)	−0.0031 (4)
N3	0.0150 (5)	0.0122 (4)	0.0138 (5)	−0.0024 (4)	0.0005 (4)	−0.0046 (4)
C1	0.0229 (6)	0.0140 (6)	0.0125 (5)	0.0003 (5)	−0.0015 (5)	0.0011 (4)
C2	0.0226 (6)	0.0123 (5)	0.0173 (6)	−0.0006 (5)	−0.0006 (5)	0.0004 (5)
C3	0.0182 (6)	0.0163 (6)	0.0114 (5)	−0.0001 (4)	0.0005 (4)	−0.0028 (4)
C4	0.0172 (6)	0.0127 (5)	0.0131 (5)	−0.0014 (4)	−0.0001 (4)	−0.0011 (4)
C5	0.0158 (5)	0.0123 (5)	0.0128 (5)	0.0014 (4)	−0.0015 (4)	−0.0018 (4)
C6	0.0159 (5)	0.0133 (5)	0.0122 (5)	0.0008 (4)	−0.0012 (4)	−0.0018 (4)
C7	0.0129 (5)	0.0123 (5)	0.0124 (5)	0.0012 (4)	−0.0020 (4)	0.0002 (4)
C8	0.0139 (5)	0.0128 (5)	0.0160 (6)	0.0008 (4)	−0.0015 (4)	−0.0016 (4)
C9	0.0180 (6)	0.0188 (6)	0.0165 (6)	0.0005 (5)	−0.0001 (5)	−0.0021 (5)
C10	0.0230 (6)	0.0194 (6)	0.0205 (6)	0.0026 (5)	−0.0022 (5)	−0.0084 (5)
C11	0.0246 (7)	0.0152 (5)	0.0245 (7)	−0.0008 (5)	−0.0006 (5)	−0.0079 (5)
C12	0.0299 (8)	0.0246 (7)	0.0234 (7)	−0.0028 (6)	0.0019 (6)	−0.0040 (6)

### Geometric parameters (Å, °)

**Table:**

S1—C11	1.7041 (14)	C3—C4	1.3898 (18)
S1—C8	1.7355 (14)	C3—H3A	0.9300
O1—C6	1.2222 (15)	C4—C5	1.3948 (19)
O2—C7	1.2367 (15)	C4—H4A	0.9300
N1—C3	1.3412 (18)	C5—C6	1.4994 (18)
N1—C2	1.3445 (18)	C7—C8	1.4736 (17)
N2—C6	1.3623 (16)	C8—C9	1.3803 (19)
N2—N3	1.3890 (14)	C9—C10	1.420 (2)
N2—H1N2	0.8600	C9—C12	1.489 (2)
N3—C7	1.3611 (17)	C10—C11	1.364 (2)
N3—H1N3	0.8600	C10—H10A	0.9300
C1—C2	1.3899 (19)	C11—H11A	0.9300
C1—C5	1.3940 (18)	C12—H12A	0.9600
C1—H1A	0.9300	C12—H12B	0.9600
C2—H2A	0.9300	C12—H12C	0.9600
			
C11—S1—C8	91.61 (7)	O1—C6—C5	123.00 (12)
C3—N1—C2	117.21 (12)	N2—C6—C5	112.71 (11)
C6—N2—N3	119.98 (11)	O2—C7—N3	121.52 (11)
C6—N2—H1N2	120.0	O2—C7—C8	122.19 (12)
N3—N2—H1N2	120.0	N3—C7—C8	116.25 (11)
C7—N3—N2	119.00 (11)	C9—C8—C7	126.96 (13)
C7—N3—H1N3	120.5	C9—C8—S1	111.48 (10)
N2—N3—H1N3	120.5	C7—C8—S1	121.53 (10)
C2—C1—C5	119.18 (12)	C8—C9—C10	111.46 (13)
C2—C1—H1A	120.4	C8—C9—C12	126.03 (14)
C5—C1—H1A	120.4	C10—C9—C12	122.50 (13)
N1—C2—C1	123.01 (13)	C11—C10—C9	113.35 (13)
N1—C2—H2A	118.5	C11—C10—H10A	123.3
C1—C2—H2A	118.5	C9—C10—H10A	123.3
N1—C3—C4	123.85 (12)	C10—C11—S1	112.10 (11)
N1—C3—H3A	118.1	C10—C11—H11A	124.0
C4—C3—H3A	118.1	S1—C11—H11A	124.0
C3—C4—C5	118.45 (12)	C9—C12—H12A	109.5
C3—C4—H4A	120.8	C9—C12—H12B	109.5
C5—C4—H4A	120.8	H12A—C12—H12B	109.5
C1—C5—C4	118.16 (12)	C9—C12—H12C	109.5
C1—C5—C6	119.18 (11)	H12A—C12—H12C	109.5
C4—C5—C6	122.64 (12)	H12B—C12—H12C	109.5
O1—C6—N2	124.29 (12)		
			
C6—N2—N3—C7	−81.27 (15)	N2—N3—C7—C8	−175.15 (11)
C3—N1—C2—C1	2.3 (2)	O2—C7—C8—C9	21.3 (2)
C5—C1—C2—N1	0.6 (2)	N3—C7—C8—C9	−161.26 (13)
C2—N1—C3—C4	−2.5 (2)	O2—C7—C8—S1	−156.60 (11)
N1—C3—C4—C5	−0.2 (2)	N3—C7—C8—S1	20.83 (16)
C2—C1—C5—C4	−3.4 (2)	C11—S1—C8—C9	−0.12 (11)
C2—C1—C5—C6	175.06 (12)	C11—S1—C8—C7	178.09 (12)
C3—C4—C5—C1	3.2 (2)	C7—C8—C9—C10	−178.07 (13)
C3—C4—C5—C6	−175.20 (12)	S1—C8—C9—C10	0.02 (15)
N3—N2—C6—O1	−6.2 (2)	C7—C8—C9—C12	3.3 (2)
N3—N2—C6—C5	173.77 (11)	S1—C8—C9—C12	−178.58 (13)
C1—C5—C6—O1	32.1 (2)	C8—C9—C10—C11	0.12 (19)
C4—C5—C6—O1	−149.49 (14)	C12—C9—C10—C11	178.78 (14)
C1—C5—C6—N2	−147.86 (13)	C9—C10—C11—S1	−0.21 (17)
C4—C5—C6—N2	30.54 (18)	C8—S1—C11—C10	0.19 (12)
N2—N3—C7—O2	2.30 (18)		

### Hydrogen-bond geometry (Å, °)

**Table:**

*D*—H···*A*	*D*—H	H···*A*	*D*···*A*	*D*—H···*A*
N2—H1N2···N1^i^	0.86	2.14	2.9068 (17)	149
N3—H1N3···O2^ii^	0.86	1.99	2.8034 (15)	158
C4—H4A···O1^iii^	0.93	2.58	3.2047 (17)	125
C10—H10A···O2^iv^	0.93	2.51	3.3928 (19)	159
C11—H11A···Cg1^v^	0.93	2.80	3.3760 (17)	121

Symmetry codes: (i) −*x*, *y*+1/2, −*z*−1/2; (ii) *x*+1/2, −*y*+3/2, −*z*; (iii) −*x*+1/2, −*y*+1, *z*−1/2; (iv) −*x*, *y*+1/2, −*z*+1/2; (v) −*x*, *y*+3/2, −*z*+1/2.
